# The Draft Regulations under the Dangerous Drugs Act

**Published:** 1921-02-19

**Authors:** S. Dunstan

**Affiliations:** Superintending Pharmacist. Royal Victoria Infirmary, Newcastle-on-Tyne


### THE EDITOR'S LETTER-BOX.

**Correspondence on all subjects is invited, but we cannot in any way be responsible for the opinions expressed by our correspondents, who must give their name and address as a guarantee of good faith, but not necessarily for publication. Correspondents are reminded that brevity of style and conciseness of statement greatly facilitate early insertion.**

### The Draft Regulations Under the Dangerous Drugs Act.

*To the Editor of* The Hospital.

Sir,—The proposed control of supply, as published some little time ago by the Home Office, appears to have aroused considerable agitation and general disapproval throughout the country, but from a hospital pharmacist's point of view I do not think there is any cause for much alarm, either on the ground of extra time or clerical work required. We have put these regulations to a practical test in this hospital, and it may be interesting to briefly describe the results of our experiment:

*Poison Cupboard No.* 1 contains all bulk poisons as they are received from the wholesale houses, also a record as to when purchased and from whom obtained, on the one side, and issues on the other.

*Poison Cupboard No.* 2 contains small quantities of poisons received from cupboard No. 1 on the one side, and issues on the other.

*Poison Cupboard No.* 3 contains all the various solutions and preparations made from the poisons received from cupboard No. 2. This cupboard also contains a record of all the solutions, such as quantities made, prepared by, and checked by, on the one side, and the date when used in the dispensary, patient's name, doctor prescribing, dispensed by, and checked on the issues side.

In practice it is found more convenient to have on the dispensary counter two sheets, one for out-patients and one for in-patients, with the following heading: Date. Poison. Quantity. Doctor. Patient. Dispensed by. Checked by.

The issues are written up at the end of the day on stock sheets in No. 3 cupboard, and checked by the stock supplies.

All ward injections or other solutions are supplied on a requisition book only, signed by the resident medical officer. This book is kept in the ward, on the one side the quantities received, and on the other side the patient's name, quantities used, whom by, and the initials of the doctor prescribing, so that these quantities used can be checked by the quantities supplied from the dispensary.

These arrangements being complete, it is found that on a week's working, dispensing on an average 2,700 prescriptions, only eighteen entries had to be made for the in-patients and ten for the out-patients, and it is estimated that the extra time required to work these regulations is not more than one hour per week.—Yours faithfully,

S. Dunstan,
Superintending Pharmacist.

Royal Victoria Infirmary, Newcastle-on-Tyne,
February 4, 1921.

